# Chitosan Micro-Grooved Membranes with Increased Asymmetry for the Improvement of the Schwann Cell Response in Nerve Regeneration

**DOI:** 10.3390/ijms22157901

**Published:** 2021-07-23

**Authors:** Luca Scaccini, Roberta Mezzena, Alessia De Masi, Mariacristina Gagliardi, Giovanna Gambarotta, Marco Cecchini, Ilaria Tonazzini

**Affiliations:** 1 National Enterprise for Nanoscience and Nanotechnology (NEST), Istituto Nanoscienze Consiglio Nazionale delle Ricerche (CNR) and Scuola Normale Superiore Pisa, Piazza San Silvestro 12, 56127 Pisa, Italy; luca.scaccini@sns.it (L.S.); roberta.mezzena@sns.it (R.M.); alessia.demasi@epfl.ch (A.D.M.); mariacristina.gagliardi@nano.cnr.it (M.G.); marco.cecchini@nano.cnr.it (M.C.); 2 Department of Clinical and Biological Sciences (DSCB), University of Torino, Orbassano, 10043 Turin, Italy; giovanna.gambarotta@unito.it; 3 Neuroscience Institute Cavalieri Ottolenghi (NICO), Regione Gonzole 10, Orbassano, 10043 Turin, Italy

**Keywords:** contact guidance, Schwann cells, nerve regeneration

## Abstract

Peripheral nerve injuries are a common condition in which a nerve is damaged, affecting more than one million people every year. There are still no efficient therapeutic treatments for these injuries. Artificial scaffolds can offer new opportunities for nerve regeneration applications; in this framework, chitosan is emerging as a promising biomaterial. Here, we set up a simple and effective method for the production of micro-structured chitosan films by solvent casting, with high fidelity in the micro-pattern reproducibility. Three types of chitosan directional micro-grooved patterns, presenting different levels of symmetricity, were developed for application in nerve regenerative medicine: gratings (GR), isosceles triangles (ISO) and scalene triangles (SCA). The directional patterns were tested with a Schwann cell line. The most asymmetric topography (SCA), although it polarized the cell shaping less efficiently, promoted higher cell proliferation and a faster cell migration, both individually and collectively, with a higher directional persistence of motion. Overall, the use of micro-structured asymmetrical directional topographies may be exploited to enhance the nerve regeneration process mediated by chitosan scaffolds.

## 1. Introduction

Peripheral nerve injuries (PNIs), a condition in which a peripheral nerve presents damage due to direct trauma or laceration [1], are a common type of injury around the world, affecting more than one million people every year, with high healthcare and social costs [2,3]. Despite the intrinsic regeneration capacity of the peripheral nervous system (PNS), the repair efficacy depends on the extent of the cut and the distance between the two nerve ends [4]. Schwann cells (SCs) are glial cells ensheathing peripheral nerve axons. In the response to nerve injuries, SCs are one of the first components involved, undergoing rapid changes in their phenotype, and proliferating and migrating from the proximal and the distal stump [5] to form the so-called Büngner bands [6]. These structures are fundamental to the regeneration process, providing a track on which neurons can regrow. Hence, a dependence of the natural healing process of peripheral nerves on the environmental topographical signals coming from the disrupted area seems to be present [7]. There are still no efficient therapeutic treatments for PNIs. The conventional direct end-to-end suturing method can only be used for peripheral nerve gaps shorter than 5 mm. When this method was used on larger gaps, it showed poor results and the amplification of the damage due to excessive tension on the affected nerve [8]. Autologous and heterologous cell transplants are not widely applied, due to the Schwann cells’ (SC) availability, and due to the safety and ethical concerns related to the in vivo use of multipotent and pluripotent human stem cells [9,10]. Nerve autografts remain the gold standard in clinics [11]. Nevertheless, this system presents its downsides. In fact, nerve tissue collection from the same patient and the need for a second surgical procedure to harvest graft tissue limit the application of this technique [12]. Artificial scaffolds can offer a new possibility in providing a fast and efficient treatment of nerve injuries. They act as a temporary extracellular matrix, providing the structural support needed in the healing process [13]. The biocompatibility of new materials, along with their biodegradability, make artificial scaffolds the perfect candidates for the effective treatment of PNIs [14].

Among the newly employed biomaterials, Chitosan—a natural chitin-derived polymer—is raising increasing interest. Chitosan is obtained via the controlled deacetylation of chitin, a major component of the shell of shrimps and other crustaceans [15]. This biomaterial is completely biocompatible, non-immunogenic and has been approved by the Food and Drug Administration for medical use [16]. Basic plain chitosan nerve conduits have been approved for clinical use in Europe, and the results showed that peripheral nerve regeneration could be improved significantly using these devices [17,18].

Recently, the use of hollow nerve guidance conduits (NGCs) started, leading to encouraging results [19]. The architectural features of NGCs are crucial [20]: many approaches have been explored to stimulate the correct positioning of the axons inside the conduit by providing topographical guiding cues. This guidance can be conveyed by filling the lumen of the conduit with appropriate biomaterials, or by enhancing the inner part of NGCs with nano and microstructures [21,22].

In fact, cells can perceive physico-mechanical stimuli from the environment at the nanometer scale (e.g., roughness, directional fibers, fractal dimensions) [23,24], regulating their behavior and fate in response to these stimuli, both in vivo and in vitro [25,26]. Several studies have demonstrated that neural cells cultured on nano/micro-structured substrates actively tune their morphology, proliferation and migration [27,28,29]. In particular, nano/micro-gratings (GRs), anisotropic topographies composed of alternating lines of grooves and ridges with lateral dimensions within 10 µm, have been extensively studied for their ability to induce directional stimuli to neural cells, in a process solely based on cell-surface contact [30]. Specifically, we demonstrated that small-period GRs in polydimethylsiloxane (PDMS) are particularly effective in driving collective SC migration and wound healing by contact guidance [31]. New evidence shows that cells can respond also to the symmetry of patterns. Interestingly, osteoblasts have been shown to modulate their shaping and migration speed in response to substrates with different levels of rotational symmetry [32].

In this work, we developed and tested in vitro chitosan-based micro-structured substrates, aiming to introduce new strategies to improve the scaffolds for peripheral nerve regeneration. Three types of chitosan directional patterns, presenting different levels of symmetry, were developed by solvent casting: gratings (GR), isosceles triangles (ISO) and scalene triangles (SCA). The chitosan topographies were then tested in vitro with the RT4-D6P2T-GFP glial Schwann cell line [33] in order to investigate their cell adhesion and proliferation, single-cell migration, and wound-healing response. Furthermore, cytoskeleton/nuclear organization and cell-cell junctions were evaluated via immunostaining for actin fibers and N-Cadherin, a protein that mediates cell–cell and cell–extracellular matrix adhesion [34].

## 2. Results

### 2.1. Chitosan Micropatterned Membranes

**Figure 1:**
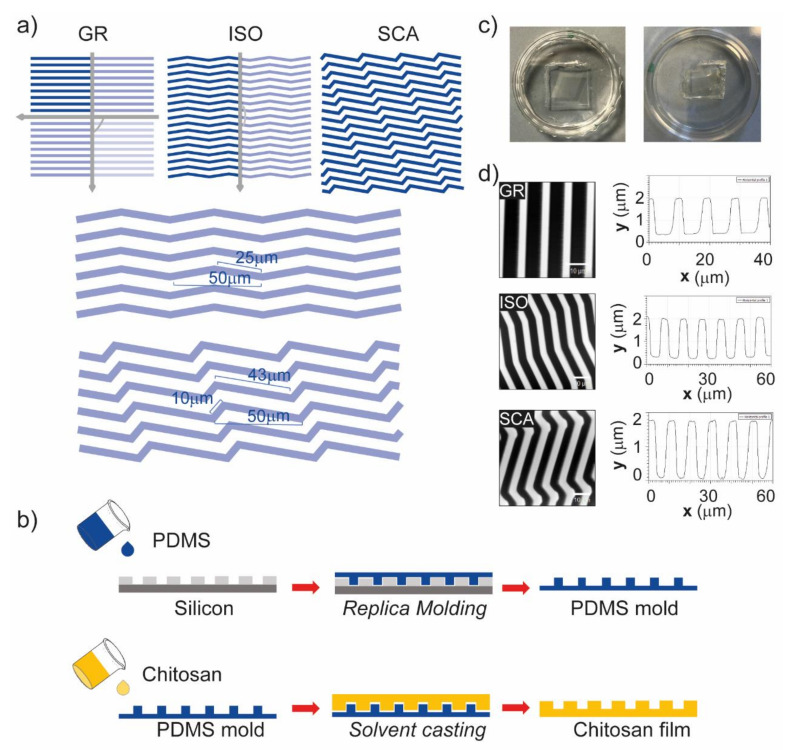
Chitosan microstructured membrane development. (**a**) CAD designs of the three patterns: gratings (GR), with two symmetry axes (grey arrows); isosceles triangles (ISO), with one symmetry axis; and scalene triangles (SCA), with no symmetry axis. All of the patterns had 4 μm ridges and 6 μm grooves; in detail, the ISO has triangles had a 50 μm base and 25 μm sides (minor angle 10°), the the SCA had triangles with a 50 μm base and 10 μm and 43 μm sides (minor angle 10°). (**b**) The microtextured chitosan membrane fabrication process with two molds by replica molding and solvent casting. (**c**) Images of a PDMS mold and a chitosan micropatterned membrane. (**d**) AFM measurements and the extracted profile of the micropatterned chitosan membranes. From the top: GR, ISO and SCA. Scale bar = 10 μm.

Chitosan membranes were microfabricated and patterned with anisotropic topographies with different levels of axial symmetry by solvent casting on PDMS intermediate molds. First of all, the patterns were written on a negative resist layer (3 μm thick) spun on a silicon wafer by laser lithography, which reads a CAD file and transfers the pattern onto the photosensitive resist using a laser. Different directional grooved patterns were designed, all based on a period (i.e., the width of a ridge and a groove) of 10 µm, with 4 µm ridges and 6 µm grooves, and a depth of ~1.6 µm. These dimensions were chosen to obtain a contact guidance mode (i.e., features with a shorter period than the cell body diameter) of interaction between the substrates and cells, according to our previous work [31]. The following directional micropatterns were developed, with different levels of symmetry (Figure 1a): a grating pattern (GR), with two symmetry axes; a zigzag pattern with isosceles triangles (ISO), with one symmetry axis; and a zigzag pattern with scalene triangles (SCA), with no symmetry axis. Regarding the triangle dimensions (side length and angles), we chose to use a linear dimension for the base of the triangles of 50 µm and an angle of 10° for both the ISO and SCA (minor angle). These values were chosen as the polarized cell somas (i.e., ≈40–50 µm long on micropatterns) can sense a full topographical feature (a triangle), and the triangles that originated were not too narrow, nor were the sides too transversal in respect to the main direction of the pattern, respectively.

These patterns were transferred onto PDMS intermediate molds. The cured PDMS was poured onto the initial silicon molds and then baked. The PDMS molds were then used to structure medium molecular weight chitosan by solvent casting (Figure 1b). A 2% *w*/*v* solution of chitosan was filtered, sonicated and poured onto the PDMS intermediate molds, and the solvent was allowed to evaporate at room temperature overnight. Then, chitosan films were peeled off of the PDMS (Figure 1c); the films dried and naturally detached from the PDMS mold, further decreasing the risk of rupture associated with the peeling-off procedure needed after the drying. Isotropic flat membranes (FLAT) were also developed as control substrates.

The topographies created on the chitosan membranes were then characterized using an atomic force microscope (AFM) (Figure 1d). The AFM measurements clearly showed that the patterns had been printed onto the chitosan with the expected period of 10 μm (with 4 μm ridges and 6 μm grooves), and the heights of the features were 1.59 ± 0.03 μm for the GR, 1.72 ± 0.01 μm for the ISO, and 1.68 ± 0.01 μm for the SCA (mean ± SD).

In order to avoid the solubilization of the chitosan films, they were processed for deacetylation in a solution of NaOH 0.5% *w*/*v* for 30 min. This concentration and time treatment allowed a non-aggressive process, leaving all of the mechanical and physical properties of the chitosan films intact (Figure 1d).

**Figure 2:**
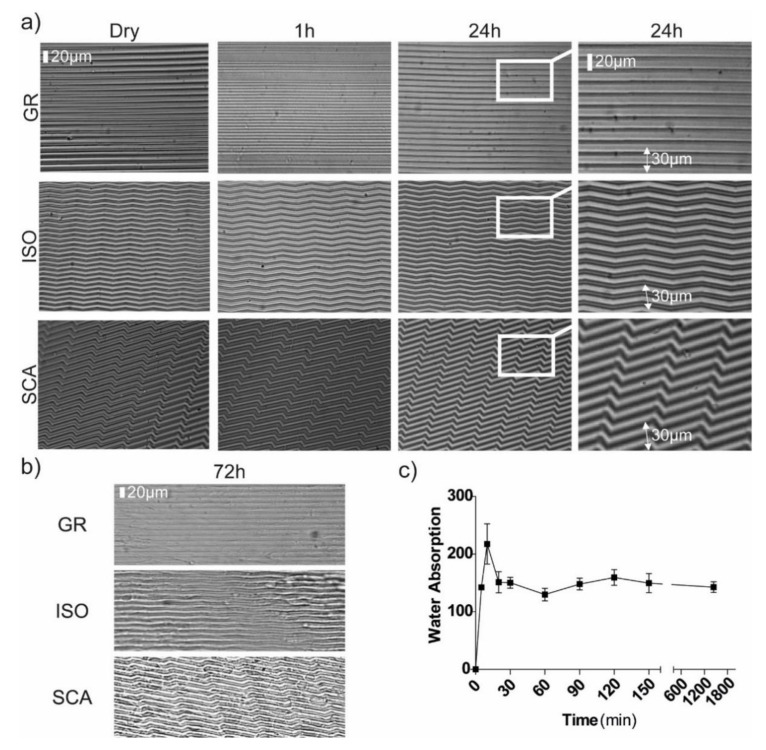
Chitosan microstructured membranes. (**a**,**b**) Optical microscope images of chitosan micropatterned membranes for the three patterns: GR, ISO, and SCA. (**a**) In a dry state (first column) and, after neutralization, in a liquid (water) environment for 1 h (second column) and 24 h (third–fourth columns). Scale bar = 20 μm. (**b**) GR, ISO and SCA membranes after 72 h in the cell culture conditions. Scale bar = 20 μm. (**c**) Water absorption of the microstructured chitosan films at different times after immersion in water. Data = mean ± SD, *n* = 5.

In fact, we checked the effects of the exposure to water, neutralization step and cell culture on our micro-structured membranes by the optical imaging of the membranes. We imaged the membranes over time and measured their features at different time-points: in the initial non-neutralized state (dry), and after neutralization at 1 and 24 h in water. The chitosan films maintained their structure and the topographical micro-features remained unaltered after the exposure to liquids (Figure 2a,b). There were no appreciable differences regarding the original dimensions of the ridges and grooves over time. Moreover, the micro-structured membranes remained stable and unchanged after several days in the cell culture conditions (Figure 2b).

Swelling is one of the most important properties of chitosan films, which characterizes their further use in biomedical applications. Therefore, we performed a water absorption test on our chitosan films, in order to measure their hydrophilicity by assessing the variation of the water absorption (WA) during the time. We evaluated the amount of water adsorbed by the membrane at different times (t = 5 min–72 h) and calculated the WA (Figure 2c). In our tests, the WA reached its maximum after 10 min: after the initial pick, the chitosan membranes reached a steady state of hydration and the WA reached an equilibrium level (around 140%), which then remained stable until 24 h.

Altogether, the previous results demonstrate that this soft lithography technique with a two-mold step allows the development of conveniently micro-grooved chitosan films. Importantly, the production process by solvent casting optimized at room temperature avoids silicon mold deterioration over time, thanks to the intermediate mold in PDMS, and it simplifies the membrane detachment. Moreover, this technique does not involve any harsh chemical or high-temperature passage. The micro-topography is well retained by the chitosan membranes after all the passages and the exposure to liquid over time. Overall, our microstructured chitosan membranes show good stability and hydrophilicity properties.

### 2.2. In Vitro Tests

In order to study the efficacy of the fabricated chitosan membranes for peripheral nerve regeneration, chitosan membranes were tested in vitro with RT4-SCs, a Schwann cell line (RT4-D6P2T) stably expressing the green-fluorescent-protein (GFP), in order to assess their ability to direct and improve Schwann cell proliferation, polarization and migration. For all of the cell experiments, the chitosan films were coated with PLL, in order to facilitate the cell attachment to the material.

**Figure 3:**
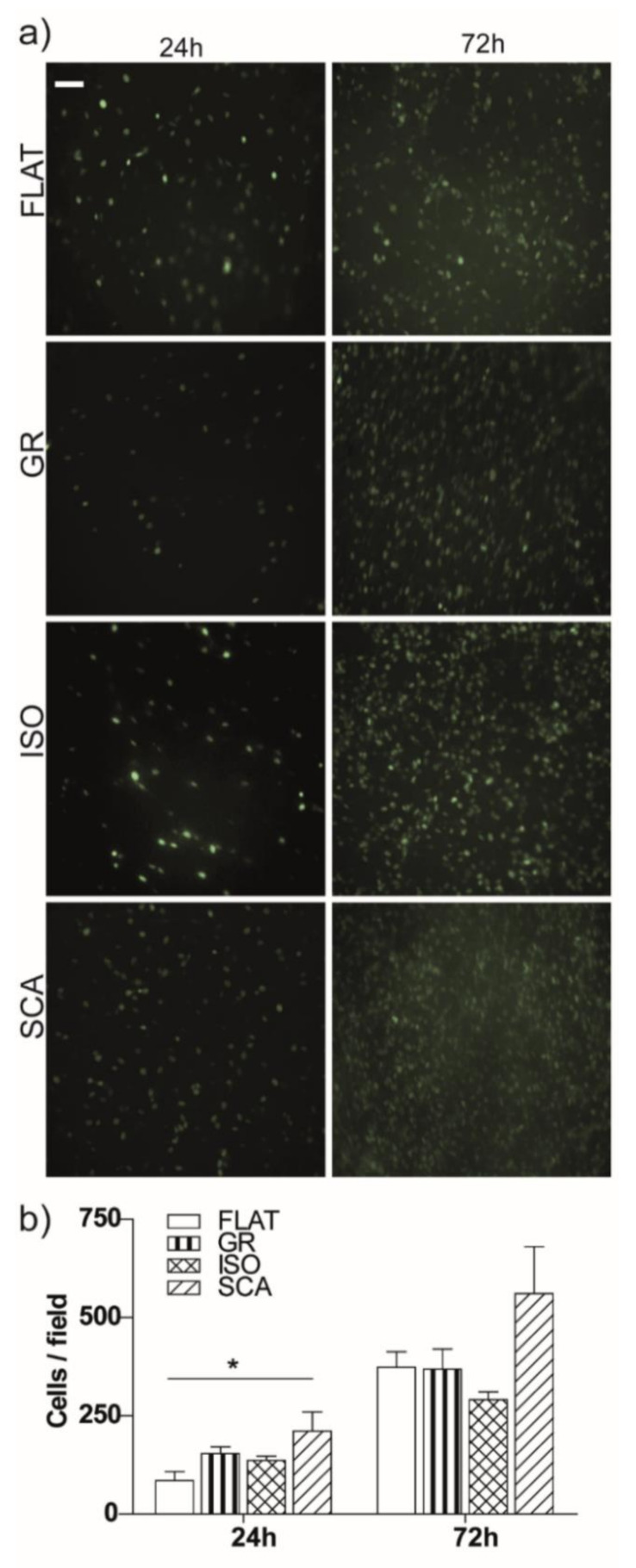
RT4-Schwann cell proliferation. (**a**) Fluorescence microscopy images of RT4-SCs at t = 24 h and t = 72 h, on FLAT, GR, ISO and SCA chitosan membranes. The nuclei are visible in green (GFP). Scale bar = 70 µm. (**b**) The proliferation rate of RT4-SCs on GR, ISO and SCA micro-structured membranes and on the FLAT control, reported as cells/field at t = 24 and 72 h. As a comparison, we report that the number of RT4-SCs on the standard cell plates (seeded in parallel and in the same conditions as the chitosan films) was 183.3 ± 63.9 cells/field at 24 h. * *p* < 0.05, one-way ANOVA, Bonferroni’s test. Data = mean ± SEM, *n* = 3.

At first, the cell proliferation on the different chitosan substrates was followed up to 72 h, using both bright-field and fluorescence microscopy, and the number of adhered cells/fields on each substrate was then quantified at t = 24 and 72 h (Figure 3). RT4-SCs attached smoothly to the membranes and rapidly started replicating. After 24 h, we found a significant enhancement in the cell amount on SCA membranes (*p* < 0.05 SCA vs. FLAT). RT4-SCs further proliferated, and after 72 h they almost reached a confluent level on the chitosan substrates. A light difference persisted between the SCA and FLAT chitosan membranes, even if it was not significant. As a further control, we also tested the cell viability using propidium iodide (PI) staining. At 72 h, we observed that the PI-positive dead cell level was similar in all of the membranes and control plates, and it was consistent with a standard cell dead turnover rate (Figure S1–Supplementary).

In general, RT4-SCs adhere and grow optimally on our micro-grooved asymmetric chitosan membranes. Moreover, the topography of the chitosan films is relevant for the adhesion and proliferation of RT4-SCs; a clear improvement in cell density is present on the SCA topography after 24 h.

### 2.3. Morphology

**Figure 4:**
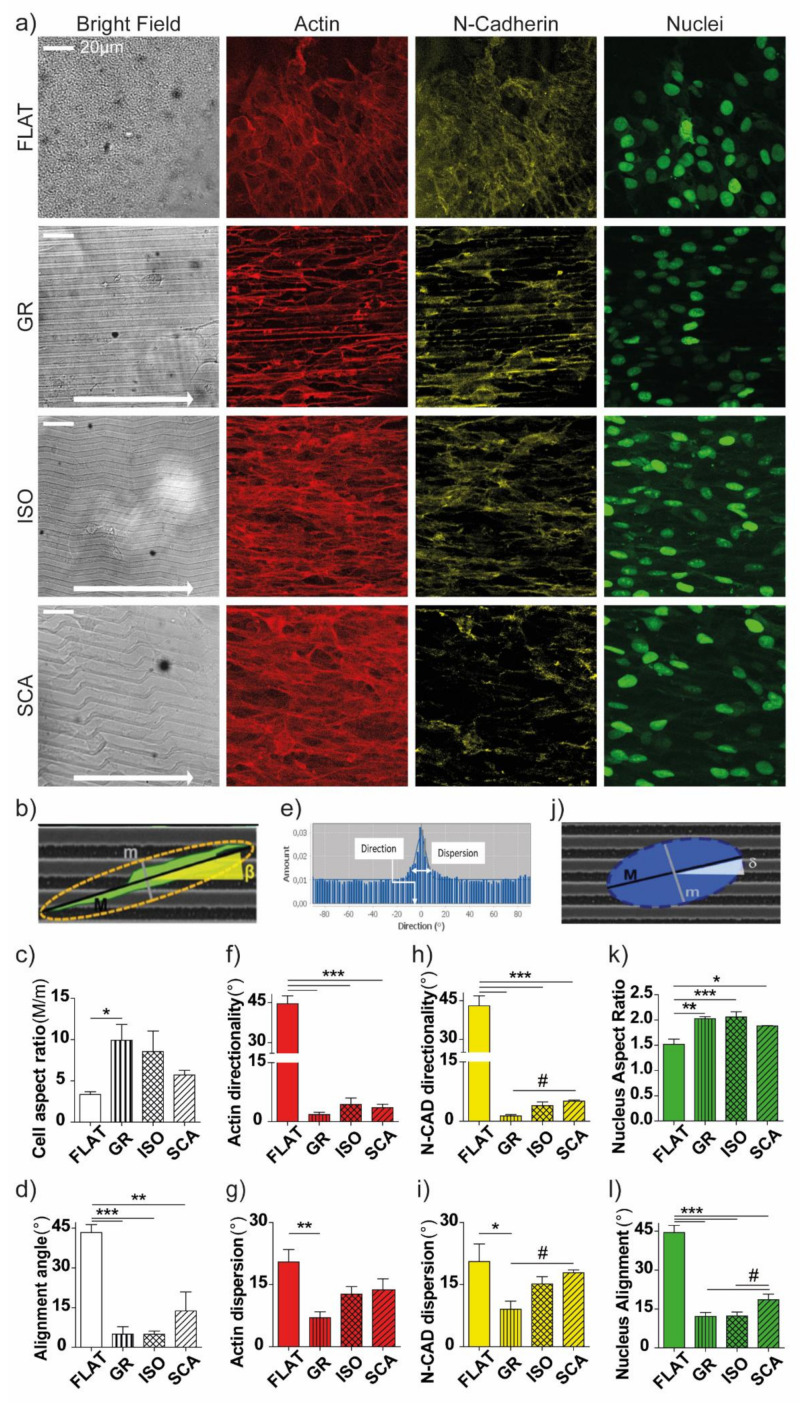
RT4-Schwann cell morphological analysis. (**a**) Confocal images of RT4-Schwann cells cultured on GR, ISO, SCA and FLAT chitosan membranes. From left to right: bright field, actin fibers (red), N-Cadherin (yellow), nuclei (green); scale bar = 20 µm. White arrows (insets in the BF images): pattern main direction (0°). (**b**–**e**) RT4-SCs’ morphological characterization. Data = mean ± SEM, *n* ≥ 3. (**b**) The single-cell morphological analysis was performed by manually tracking each cell shape (on actin fiber images): (**c**) Cell aspect ratio: the ratio of the length of the major axis M to the minor axis m of the best-fitted ellipse on the drawn cell shape. (**d**) Cell alignment angle: the angle (β) between the cell major axis and the main axes of the pattern. A random direction was chosen for the FLAT. (**e**) Representative fast Fourier transform (FFT) analysis, returning the directionality and dispersion values; (**f**,**g**) actin fiber cytoskeleton organization analysis: the actin directionality (**f**) and dispersion (**g**) of the RT4-SCs on GR, ISO and SCA micro-structured membranes, and on the FLAT control. (**h**,**i**) Analysis of the N-Cad positive cell–cell junctions’ organization: N-Cad directionality (**h**) and dispersion (**i**) for RT4-SCs cultured on GR, ISO and SCA micro-structured membranes, and on the FLAT control. (**j**) Nuclear morphological analysis: (**k**) nucleus aspect ratio (AR, nuclear major axis/minor axis) and (**l**) nucleus alignment angle (δ) of the RT4-SCs on the GR, ISO and SCA micro-structured membranes, and on the FLAT control. * *p* < 0.05, ** *p* < 0.001, *** *p* < 0.0001, one-way ANOVA, Bonferroni’s test; # *p <* 0.05 Bonferroni’s selected test. Data = mean ± SEM, *n* ≥ 3.

We also performed a morphological analysis on the RT4-SCs cultured on our micro-structured chitosan surfaces (at 72 h). The cells were processed for immunostaining with phalloidin (actin fibers marker) and an anti-N-Cadherin antibody (N-Cad, the cell junction’s marker) (Figure 4a; merged channels in Figure S2–Supplementary).

We analyzed the response of individual RT4-SCs by measuring the cell shape (elongation ratio) and cell-body alignment to the pattern (Figure 4b–d). The cell spreading area was the same for all of the topographies (1540 ± 267 µm^2^, 1814 ± 133 µm^2^, 1737 ± 150 µm^2^, 1447 ± 230 µm^2^ on FLAT, GR, ISO, SCA, respectively). The RT4-SCs cultured on GR showed increased elongation (*p* < 0.05 FLAT vs. GR), but their elongation showed a decreasing trend on the most asymmetric patterns (Figure 4c). RT4-SCs aligned to all of the patterns, with an average alignment angle below ~5° on GR and ISO (*p* < 0.001 FLAT vs. GR and ISO), and of ~14° on SCA (*p* < 0.01 FLAT vs. SCA) (Figure 4d). Overall, GR highly polarizes and aligns single RT4-SCs, while SCA shows moderate results in this framework.

We then analyzed the organization of the cytoskeleton in the RT4-SC layers on our chitosan membranes, looking first at the directionality of the actin fibers. The actin orientation was quantified by the Fast Fourier Transform (FFT) analysis of the fluorescent images, which returns the actin fibers’ signal dispersion (i.e., the indicator for the angular spread of the actin fluorescence signal) and directionality (an indicator for the overall degree of orientation of the cytoskeleton fibers, here normalized against the underlying pattern direction) (Figure 4e). As expected, all of the patterns were highly efficient in orientating the RT4-SC cytoskeleton, with an average directionality of actin fibers below 5° in respect to the pattern orientation (*p* < 0.0001, FLAT vs. GR/ISO/SCA), while on the FLAT control the angular distribution covered the entire range of angles, with the expected average angle being around 45° (Figure 4f). Regarding the dispersion of the actin signal, only GR decreased significantly the dispersion of the actin signal (*p* < 0.01, FLAT vs. GR), by highly polarizing the fibers along the GR. However, even if no significant differences were present, a clear trend of increasing dispersion with the increase of the pattern asymmetry was visible (Figure 4g).

The N-Cad expression pattern, as a marker of cell–cell contact, was then analyzed using the same method presented for the actin. The localization of the N-Cad developed was oriented and aligned to the main axis of the topographies, while it appeared to be randomly organized on the FLAT. We found that all of the directional patterns (GR, ISO and SCA) guided the N-Cad orientation (*p* < 0.001 FLAT vs. GR/ISO/SCA, Figure 4h) for the cytoskeleton fibers’ organization. Regarding the dispersion of the N-Cad signal, only GR highly polarized the N-Cad signal, and no significant differences between the asymmetric ISO and SCA patterns emerged with respect to the isotropic FLAT substrate (Figure 4i). In particular, the dispersion of the N-Cad signal was significantly higher on the SCA with respect to the GR (*p* < 0.05 GR vs. SCA).

Overall, these data demonstrate that all of the topographies are effective in inducing cell polarization, tuning actin organization and cell–cell contacts along the pattern main direction. The GR presents a lower dispersion of the fluorescent markers signals, while the more asymmetric SCA polarizes and guides RT4-SCs less strictly, showing a higher dispersion of the signals. Altogether, these data show that RT4-SCs are guided by the underlying directional micro-topographies, but are also shaped according to their asymmetry. These effects become more pronounced as the asymmetry of the pattern increases.

Because the nuclear reorientation can determine the cell polarity and migration [35], we also investigated the morphological parameters of the nuclei (Figure 4j–l). We quantified two nuclear shape-defining parameters: the aspect ratio (the ratio of its major axis to its minor axis) and the alignment angle (°, in respect to the pattern orientation). The nuclear AR changed if the cells were cultured on different patterns. On FLAT films, the nuclei were more rounded, and they were more elongated on the GR and ISO, showing here a higher nuclear AR (*p* < 0.001 FLAT vs. GR and ISO) and, even if at a little extent, also on SCA (*p* < 0.05 FLAT vs. SCA) (Figure 4k). Concerning the nuclear alignment, the nuclei stretched following the main direction of the patterns for all of them (*p* < 0.001 FLAT vs. all, Figure 4l) even if here, again, they showed a lower nuclear alignment on SCA (*p* < 0.05 SCA vs. GR and vs. ISO, # Bonferroni’s selected tests).

As for the previous assays, the differences were raised between the different micro-structures: again, the SCA topography leaves a higher degree of freedom for the nuclear orientation in the RT4-SCs.

### 2.4. Cell Migration

**Figure 5:**
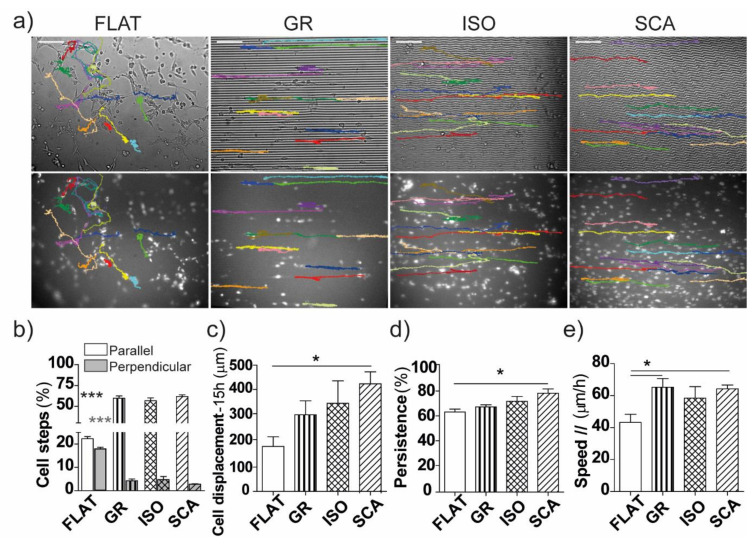
Single cell migration. (**a**) Representative bright-field images of the RT4-SC migration patterns (colored tracks) on FLAT, GR, ISO and SCA, in the bright field (first row) and in GFP-fluorescence (second row). The migration was followed by time-lapse microscopy for 20 h; scale bar = 100 µm. (**b**–**e**) Single cell migration analysis (data = mean ± SEM, *n* ≥ 3): (**b**) percentage of steps parallel (±15° in respect to the pattern’s main direction; white columns) and perpendicular (±75–105° to the pattern’s main direction; grey columns) to the pattern’s orientation for each substrate: *** *p* < 0.001 FLAT vs. all micropatterns, one-way ANOVA, Bonferroni’s test. (**c**) The final cell displacement (at t = 15 h) from the t = 0 position, on different substrates: * *p* < 0.05 SCA vs. FLAT, one-way ANOVA, Bonferroni’s test. (**d**) Persistence: the percentage of parallel steps in the same direction of migration: * *p* < 0.05 SCA vs. FLAT, one-way ANOVA. Bonferroni’s test. (**e**) The average cell speed (µm/h) in the parallel direction (±15° in respect to pattern’s main direction): * *p* < 0.05 vs. FLAT, one-way ANOVA, Bonferroni’s test.

The ability of SCs to migrate along the chitosan patterns was assessed first by single-cell migration experiments. RT4-SCs were cultured on FLAT, GR, ISO and SCA chitosan films, and their motion was tracked for 20 h by time-lapse microscopy (Figure 5a). The RT4 cells migrated parallel to the main axes of all of the topographies, whereas they maintained a random walk on the FLAT membranes. The percentage of parallel steps increased in all of the chitosan patterns (Figure 5b), from 22 ± 1% for FLAT (for a random motion with an arbitrary reference direction, the percentage of the aligned steps would theoretically be 20%) to 60 ± 2% for GR, 57 ± 3% for ISO and 62 ± 2% for SCA (*p* < 0.001 all vs. FLAT). Conversely, there was a decrease in the quantity of perpendicular steps below 5% on all of the patterns (*p* < 0.001 all vs. FLAT; Figure 5b, grey columns). The decrease of symmetry in the substrate topography had a positive impact on the total displacement of the cells, as taken after 15 h of migration (Figure 5c). Generally, the topographies had a positive effect on the final displacement, which originated by the anisotropic directional migration along the patterns, and interestingly the cells on the SCA had the highest displacement (422 ± 46 µm; *p* < 0.05 SCA vs. FLAT). Indeed, the distance travelled showed an increasing trend proportional to the increase of the asymmetry in the pattern.

The level of symmetry in the pattern also had an interesting effect on the persistence of the cell motion along one direction of its migration path, considering that in this case the steps walked parallel to the pattern axes (Figure 5d). The RT4-SCs on SCA persisted for more time on one specific direction (79.6 ± 3.4% steps; *p* < 0.05 SCA vs. FLAT) and were less likely to revert their migration direction.

The mean overall migration speed was nearly the same on the FLAT (V = 44.4 ± 6.9 µm/h) and chitosan patterned membranes (V = 55.7 ± 5.1, 49.6 ± 7.2, 55.9 ± 3.4 µm/h, on GR, ISO and SCA, respectively). In order to further describe the directional nature of the single-cell migration, we selectively analyzed the velocity in its perpendicular and parallel components (V⊥ and V║, respectively). The parallel speed was roughly increased on the GR, ISO and SCA compared to FLAT, and the cells were significantly faster along the GR and SCA patterns (Figure 5e). Moreover, the speed along the perpendicular direction was significantly inhibited on the patterned samples (Figure S3–Supplementary), indicating that cells are not only less likely to move in that direction, but also slower in that particular direction (i.e., perpendicular ±15°) of migration.

Altogether, the results of the single-cell migration experiments showed that all of the micropatterns enhanced the migration (in terms of parallel steps and speed) along the main axis direction, and inhibited the deviation from it. The best-performing pattern was SCA overall, which promotes a farther displacement of the cells and an increased persistence in one direction of migration.

**Figure 6:**
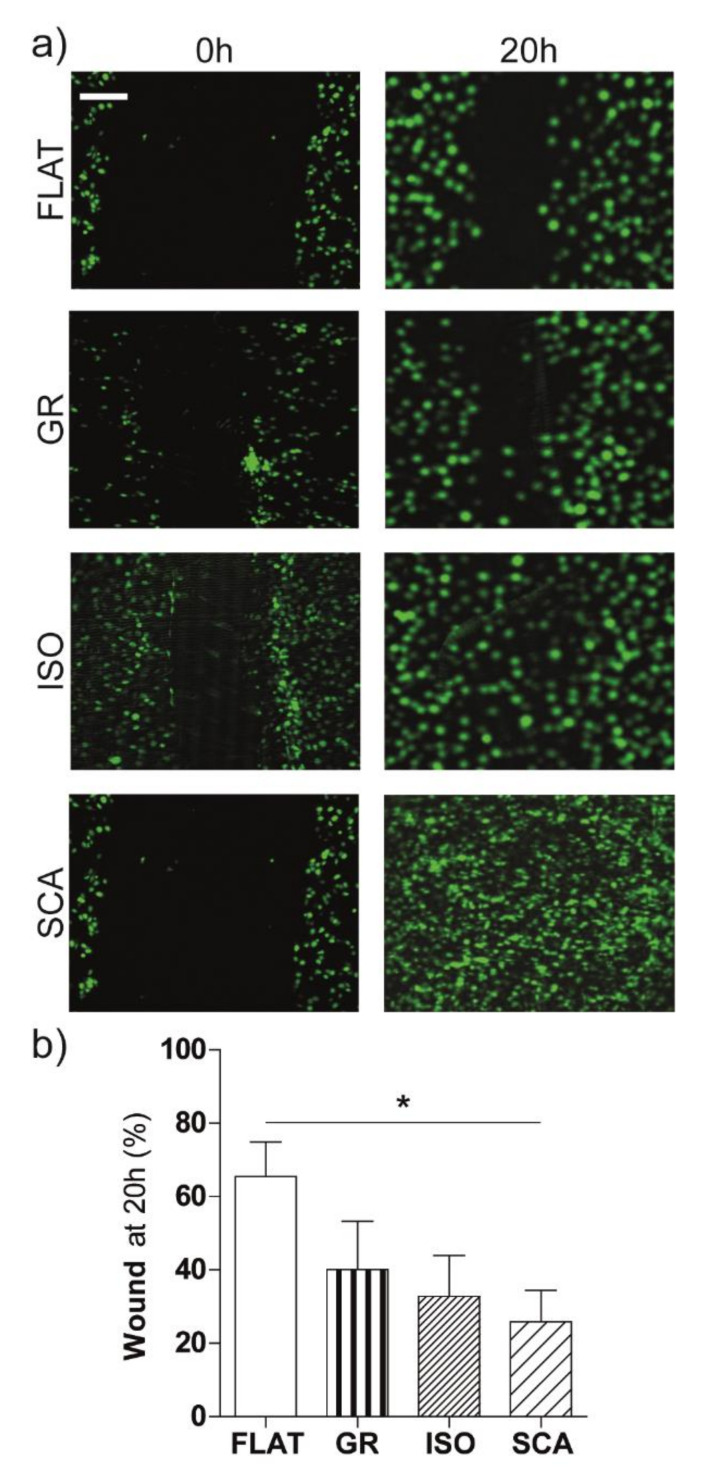
Collective cell migration. (**a**) Fluorescence microscopy images of the wound-healing experiments at t = 0 h and t = 20 h from the gap opening. The RT4-SC nuclei are visible in green (GFP) on the micro-structured chitosan films (GR, ISO and SCA) and on the FLAT control; scale bar = 100 µm. (**b**) Wound-healing quantification: the percentage of the wound area at t = 20 h was reported as a % in respect to the initial wound area (t = 0 h). * *p* < 0.05 FLAT vs. SCA, one-way ANOVA, Bonferroni’s test. Data = mean ± SEM, *n* ≥ 3.

We then performed wound-healing experiments, in order to observe how a uniform monolayer of RT4-SCs migrate on the differently symmetrical directional patterns. These experiments consisted in the generation of a cell-free area (a wound, placed perpendicular to the pattern direction) surrounded by a monolayer of cells, in order to measure the ability of the cells to re-colonize this gap and, eventually, the completely close the wound (Figure 6a). The cell migration was then observed via time-lapse microscopy, and the wound area at t = 20 h was analyzed and reported as the percentage of the wound area present in respect to the initial (t = 0) wound area. The initial gap mean width was 363 ± 34 µm for GR, 379 ± 48 µm for ISO, 410 ± 39 µm for SCA and 396 ± 28 µm for FLAT, thus ensuring the same starting conditions for all of the patterns.

In general, the RT4-Schwann cells started to migrate quickly inside the cell-free area. The micro-structured membranes showed, overall, a faster collective migration compared to the FLAT isotropic membranes used as the control. The wound area on the directional patterned substrates, after 20 h, decreased in accordance with the increase of their asymmetry. In particular, the wound area was significantly reduced on SCA (*p* < 0.05 SCA vs. FLAT, Figure 6b). As a comparison, we report that the wound area on standard cell plate (seeded in parallel and in the same conditions as the chitosan films) was 64 ± 1% after 20 h, almost the same as what was observed on the FLAT chitosan membranes.

Here again, SCA—the most asymmetric directional pattern—produces a significant enhancement in the collective cell migration. SCA is effective in directing and improving both single and collective SC migration.

### 2.5. Cell Protrusions’ Asymmetry

**Figure 7:**
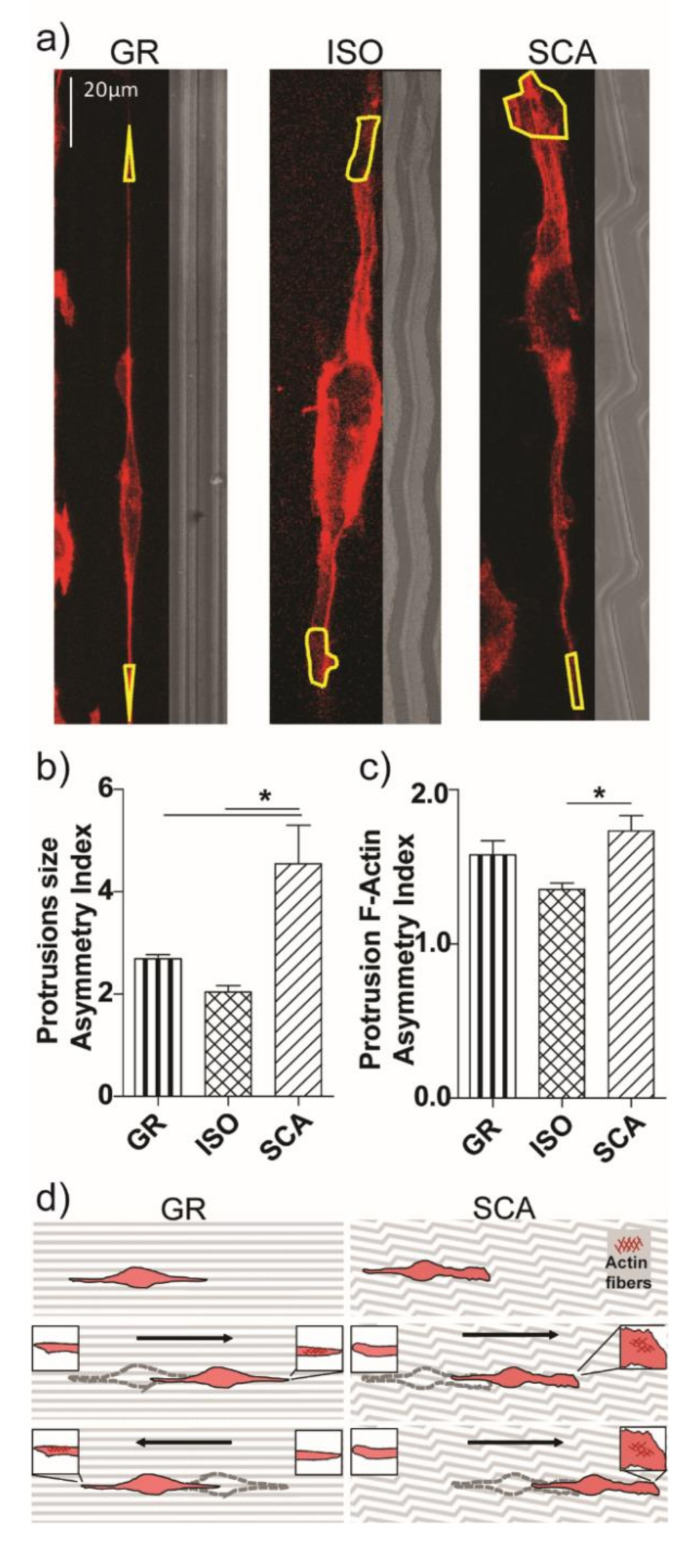
Cell asymmetry on SCA membranes. (**a**) Confocal representative images of single RT4-SCs cultured on GR, ISO, SCA chitosan membranes and stained for actin fibers (red), with the protrusion area drawn in yellow; scale bar = 20 µm. (**b**) Cell protrusion size asymmetry index: the ratio of the protrusions’ area (the ratio between the area of the bigger cell’s protrusion over the minor one), as a parameter of the cell-end asymmetry; * *p* < 0.05 SCA vs. GR and ISO, one-way ANOVA, Bonferroni’s test. (**c**) Cell protrusion F-Actin asymmetry index: the ratio of the F-actin intensity between the two cell protrusions (i.e., the F-actin mean intensity of the bigger protrusion and the minor one, for each cell), as a parameter of the cell-end contractility asymmetry; * *p* < 0.05 SCA vs. ISO, one-way ANOVA, Bonferroni’s test. Data = mean ± SEM, *n* ≥ 3. (**d**) Model of the asymmetric development and directional migration of RT4-SCs on SCA chitosan substrates. Cell–substrate interactions modulate the actin cytoskeleton organization and fiber formation (inset: dark-red mashes) and control the direction of migration. The symmetry of the pattern influences these processes, promoting or demoting differently the spreading dynamics at the two cell edges on GR or SCA. The black arrows indicate the cell migration direction.

We performed an analysis of the terminal protrusions of the RT4-SCs on our micro-structured chitosan surfaces (at 72 h) by measuring the ratio between the two cell protrusions’ areas (Figure 7a). The RT4-SCs were bipolarized on all of the microgrooved membranes, but adopted different morphologies on the patterns. The two main cytoplasmatic protrusions extend equally along GR, on both sides of the cells, resulting in a similar extension and area of both ends. We quantified this cell-end size asymmetry index in Figure 7b by calculating the ratio between the bigger side and the minor one of each cell (the ratio is therefore always > 1, even if the two sides have similar cell-end areas).

Similarly, it happened to RT4-SCs on ISO. The symmetry of the two cytoplasmic protrusions was, instead, limited on the cells growing on SCA, where one cell end was consistently larger than the other: this asymmetry led to an increase in the area ratio between the two protrusion edges (*p* < 0.05 SCA vs. ISO and GR; Figure 7b).

We then measured the cell contractility at the two main cell edges by quantifying the intensity of the actin fibers there, with the aim of showing whether an asymmetric cell protrusion profile also leads to a different cytoskeleton contractility. We analyzed the ratio of the F-actin intensity signal between the two cell-ends for each cell, e.g., an index of the protrusion of F-actin contractility asymmetry (Figure 7c). In general, the intensity of the F-actin was never equal between the two protrusions of a polarized cell, in any of the patterns. However, we found a significant increase in the Protrusion F-Actin asymmetry index of RT4-SCs on SCA (*p* < 0.05 SCA vs. ISO; Figure 7c).

Overall, SCA induces the establishment of asymmetric cell fronts in RT4-SCs, caused by the local topographical differences encountered by each cytoplasmatic protrusion during the cell spreading and migration. The asymmetric protrusion organization on SCA is also reflected in the asymmetric amount of cytoskeletal actin fibers between the cell edges. These data indicate the presence of an asymmetric cell contractility status of the RT4-Schwann cells on the SCA patterns, which likely causes the higher cell migration and directional persistence on SCA (Figure 7d).

## 3. Discussion

In this work, we have set up an optimal protocol for the production of micro-structured chitosan films by solvent casting, with considerably reduced costs in terms of the time and materials used in the process. The films presented precise and stable directional and asymmetric micro-topographies, preserving the chitosan’s biocompatibility. RT4 Schwannoma cells grew optimally on our micro-grooved chitosan membranes. All of the topographies (GR, ISO, SCA) were effective in inducing cell and nuclear alignment to the pattern, tuning the actin organization and cell–cell contacts, and guiding the cell migration along the patterns’ main direction. The data demonstrated that the RT4-SCs were guided by the underlying directional micro-topographies, but they also behaved according to the patterns’ asymmetry. The most asymmetric directional pattern, the SCA, polarized and aligned the RT4-SCs less strictly, leaving a higher degree of freedom for the nuclear/cell body orientation, and, in parallel, allowing enhanced cell adhesion and proliferation. Importantly, the SCA promoted a farther displacement of the cells and an increased persistence on one direction of migration by inducing the establishment of asymmetric cell fronts with different actin fiber organizations. Overall, the SCA chitosan membranes showed a promising topography for the improvement of SCs’ migration performance, and therefore of nerve regeneration scaffolds.

Chitosan, being an abundant and cheap natural polymer, appeared to be an interesting material for regenerative medicine, in particular for nerve tissue engineering [36,37,38,39]. Here, chitosan solvent casting was able to replicate and retain the micro-pattern features in all of the three geometries (GR, ISO, SCA), with an optimal resolution at the micrometer level. Our soft-lithography process preserved the properties of the material, avoiding its alteration and bypassing the use of toxic chemicals which could have impacted on its biocompatibility, or high temperatures (being entirely performed at room temperature). The two-mold process allowed us: (1) to avoid the deterioration of the original silicon molds over time (i.e., the most expensive step of the membrane development, which needs the use of clean-room facilities); and (2) to better detach the chitosan films from the PDMS mold, as PDMS has a very poor interaction with chitosan.

With this process, we obtained reproducible, transparent, mechanically-compliant [40] and biocompatible micro-structured chitosan membranes with a cost-effective process. Our films were highly durable over time and suitable for cell cultures. Further functionalization with bioactive compounds (e.g., neuregulin1 [24]), which are often thermo-sensible, can be easily implemented with our room-temperature protocol, by adding any water-soluble compound to the chitosan solution during the production process.

Chitosan has already shown good performances for the fabrication of neural conduits [41], but chitosan nerve guide conduits could be further improved with micro-structuration in order to enhance their regeneration potential.

Overall, the directional and most asymmetrical micro-topography SCA was shown to be the best substrate in promoting cell migration, together with cell adhesion/proliferation, even if it was the less efficient at polarizing cells at the soma, cytoskeleton, and nuclear levels. GR, presenting the more symmetrical directional pattern, promoted cell alignment with a precise highly-polarized bipolar morphology for cytoskeleton, soma and nuclear organization. SCA, instead, allowed cells to maintain a higher degree of freedom in their spreading, with a less elongated/bipolar shape and a slightly lower alignment to the pattern. ISO presented an intermediate level of symmetry between GR and SCA, and—in line with this—presented median orientation values for cell morphology.

All the patterns enhanced the cell migration (in terms of steps and speed) along one direction and inhibited the deviation from it. Even though the cells were freer to spread on the SCA, they showed here a highly directional migration, even with better performance in terms of displacement and directional persistence on one direction of migration. Importantly, these features are fundamental in peripheral nerve regeneration, where Schwann cells are required to follow a precise direction in order to fill the gap and reconnect the two nerve stumps.

Considering the migration results along with the morphology analysis, the higher degree of freedom in the shaping could be the cause for the higher motility of cells. Interestingly, this improvement in the cell migration response on asymmetric patterns is in agreement with the few previous works on substrates with different levels of rotational symmetry [32,42]. In [32], mouse osteoblastic cells (MC3T3) moved faster on PDMS semi-circular arcs (the most asymmetrical pattern) than on gratings and squares, and also showed the strongest directional persistence. This enhanced motility was not associated with the detected number of focal adhesion sites in the cells; however, the pattern asymmetry was reflected in the asymmetrical cell spreading (i.e., cytoplasmatic protrusions); although, on squares, the cells could extend equally in all directions, this freedom was limited on gratings and arcs, where the cells were polarized with two main cytoplasmatic protrusions. However, the cells on arcs had one end consistently larger than the other, resulting in a faster cell movement in this direction. The asymmetry in the cell shape determined the direction and speed of the cell migration. Another study [42] showed similar results. Osteoblastic cells (MC3T3) moved faster on PDMS zig-zag patterns with obtuse bends (angle 135°) than with acute bends (45°). Here, a correlation between the bending angle of the zig-zag pattern and the speed of the cell migration was found, and incidentally these substrates were very similar in shape to our SCA and ISO patterns, respectively. Similarly, our cells on SCA showed a higher asymmetric shaping: they were mainly bipolar, with the front of the cell having a larger cytoplasmic protrusion, as shown in Figure 7c. The two protruding fronts of the RT4-SCs on SCA showed different local topography due to the asymmetrical nature of the pattern. Moreover, the asymmetric protrusion organization on SCA is also reflected also in the amount of cytoskeletal actin fibers present at the cell edges. As a result, one end will spread more than the other, leading to an asymmetric cell shape and actin fiber formation at the cell edges. This difference allows higher cell mobility and, once the initial direction is established, a more persistent directional migration over a long period of time (Figure 7d).

Finally, it is known that the mechanical properties of the nucleus and its mechanical coupling to the plasma membrane via the cytoskeleton are also crucial in the migration and in the polarization of the cells [35]. In our experiments, the topographies played an important role in the nuclear morphology. GR and ISO were almost equally effective in stretching and aligning the nuclei to the pattern, while SCA impaired both the nuclear elongation and the alignment. Again, the SCA pattern’s asymmetry is reflected in the nuclear morphology, thus leaving a greater freedom for the nuclear shaping, such as for cells. The nuclear shaping also impacts the gene expression and epigenetic markers [43], likely further contributing to the cell behavior on the different topographies.

Overall, the ability of anisotropic asymmetric substrates to tune neural cells can be useful in peripheral nerve system regeneration frameworks, as devices patterned with directional and asymmetrical micro-topographies could be used to orientate cells in the right direction, and to achieve the more rapid and effective repair of the injured nerve.

## 4. Materials and Methods

### 4.1. Micro-Patterned Mold and Polymethilsiloxane Intermediate Mold Development

The silicon molds were micropatterned using a photolithography technique. Briefly, a wafer of silicon (Siegert Wafer) was cut into pieces with an area of 3 cm^2^. The silicon surface was pretreated with oxygen plasma exposure (Diener Electronic, Plasma Surface Technology, Ebhausen, Germany) at 100 Watt for 1 min. The negative resist SU-8:2002 (Kayaku Advanced Materials, Westborough, MA, USA) was spun at 3000 rpm for 1 min to obtain a 3 μm thickness. The coated silicon pieces were pre-backed at 95 °C for 2 min on a hot plate.

The following directional micro-patterns were developed: a grating pattern (GR), with alternating lines of grooves and ridges; a zigzag pattern with isosceles triangles (ISO); and a zigzag pattern with scalene triangles (SCA). The GR, ISO and SCA patterns were designed in KLayout software with a period of 10 μm, 4 μm ridges and 6 μm grooves, and depth of 1.6 µm. The ISO triangles had a 50 μm base and 25 μm sides (minor angle 10°), and the SCA triangles had a 50 μm base and 10 and 43 μm sides (minor angle 10°); all of the patterns were written in a 1 cm^2^ area. The FLAT control pattern was an area of 1 cm^2^, and completely exposed.

The exposure was carried out with the direct-write photolithography machine MicroWriter ML^®^3 (Durham Magneto Optics, Cambridge, UK) at a resolution of 0.6 μm and a wavelength of 385 nm. After the exposure, the samples were post-baked at 95 °C for 2 min. The samples were then developed by immersion in the SU-8 developer (Kayaku Advanced Materials, USA). The substrates were then rinsed in isopropyl alcohol (CAS n. 67-63-0, LiChrosolv, Sigma-Aldrich, St. Louis, MO, USA) and dried with a gentle nitrogen stream. The molds were hard-baked at 200 °C for 20 min.

The micro-patterned silicon molds were silanized. We used three different solutions/solvents for our silanization process: Silanization Solution I (85126, Fluka, Thermo Fisher), cyclohexane (Sigma-Aldrich, Cat No. 227048) and 1-octanol (Sigma-Aldrich, Cat No. 297887). The molds were kept in each solution (with the order described above) for 10 min, and then washed with isopropyl alcohol (IPA) and dried for 10 min at 80 °C, before proceeding with the next solvent.

Finally, polydimethylsiloxane (PDMS) intermediate mold replicas were realized with the silanized silicon molds. PDMS pre-polymer (Sylgard 184, The Dow Chemical Company, Pittsburg, CA, USA) was mixed with the curing agent (silicone elastomer curing agent, 184, Sylgard) at a ratio of 10:1 *w*/*w*, and the mixture was degassed to remove air bubbles. The cured PDMS was poured onto the silanized silicon molds and then baked at 80 °C for 90 min in the oven, to speed up the cross-linking process. The PDMS intermediate replicas were carefully detached from the silicon molds and used for the fabrication of the chitosan films through a solvent casting technique.

### 4.2. Chitosan Scaffold Fabrication

The chitosan solution, 2% *w*/*v*, was prepared using chitosan medium weight (MW) powder (Sigma-Aldrich, 448877; 75–85% deacetylation degree) dissolved in acetic acid solution (1% *v/v* in deionized (DI) water). The solution was stirred overnight at room temperature (RT) until complete dissolution. The obtained solution was filtered using a Büchner funnel with Perfecte2 filter paper with a 10 μm cut-off (Superfiltro Milano, Milan, Italy) and a vacuum pump, slowly bringing the system to a low pressure (around 250–300 mbar). This solution was stocked at 4 °C for multiple preparations of the chitosan films.

The chitosan solution was sonicated for 15 min just before its use, and was poured onto the patterned area of the PDMS mold and placed in a 35 mm Petri dish. For each mold (total size around 8 cm^2^), 0.5 g chitosan solution was used. The molds covered with chitosan solution were kept overnight under a chemical hood at RT, until the films had completely dried.

The films were neutralized for 30 min in a 0.5% *w*/*v* NaOH solution (0.5 g NaOH in 100 mL DI-water) and washed once with a phosphate buffered solution (PBS; Sigma-Aldrich, D8537). Before the cell culturing, the membranes were sterilized by treatment with ethanol 70% (for 15 min) and then rinsed with sterile H_2_O Milli-Q. The chitosan membranes were then coated with a solution of Poly-L-Lysine (PLL 0.007% in water, at RT for 30 min; Sigma-Aldrich, P4832) for proper cell adhesion.

### 4.3. Microtopography Characterization by Atomic Force Microscope

The microstructured chitosan films, with the GR, ISO and SCA patterns, were analyzed to ensure the correct transfer of the pattern from the molds to the chitosan substrates. The features of the topographies were quantitatively measured with an Atomic Force Microscope (AFM) (Veeco Innova Scanning Probe Microscope, Veeco Instruments Inc., Santa Barbara, CA, USA), operating in tapping mode. The measurements were performed in air at RT, and the scan data were leveled by surface subtraction. The data were analyzed with Gwiddion software (Gwiddion 2.47 version, Brno, Czech Republic, “Profile” tool) and reported as the mean ± standard deviation (SD) of at least 3 feature measurements for each topography type.

### 4.4. Water Absorption Test

The dried chitosan films were soaked in deionized (DI) water at RT until swelling equilibrium was obtained, for a fixed period, and were weighted at different times (t = 5 min–3 h) in order to evaluate the amount of water adsorbed by the membrane. The weight of the swollen sample (*Ws*) was measured after removing the surface water with blotting paper. The water absorption (*WA*) was then calculated on the basis of the weight of the swollen (*Ws*) and dry (*Wd*) films using the following Equation (1):

(1) $$WA=\left[\left(\frac{Ws-Wd}{Wd}\right)\right]*100,$$

The weight was measured in mg. The data are reported as the mean ± SD, *n* = 5. We also performed this measurement with membranes dried at 37 °C for at least 5 h (*n* = 2), and the data were comparable (data not shown).

### 4.5. Schwann Cell Line Culture

For the cell–material interaction experiments, the RT4-D6P2T cell line (ATCC, Manassas, VA, USA, CRL-2768), a Schwannoma cell line (SCs) derived from an N-ethyl-N-nitrosourea induced rat peripheral neuro-tumor (ATCC CRL-2768), was used [44]. These cells were transduced with a lentivirus in order to stably express Green Fluorescent Protein (GFP), as previously described [33].

RT4-D6P2T stably expressing GFP (hereinafter referred to as RT4-SCs) were cultured and maintained at 37 °C in a humidified atmosphere of 5% CO_2_/air, in Dulbecco’s modified Eagle’s medium (DMEM) supplemented with 4.5 g/L glucose, 100 U/mL penicillin, 100 μg/mL streptomycin, 0.11 g/L sodium pyruvate, 4 mM L-glutamine and 10% heat-inactivated fetal bovine serum (FBS). All of the products were from Thermo-Fisher Scientific (Waltham, MA, USA).

### 4.6. Cell Proliferation Assay

RT4-SCs cells were seeded on chitosan membranes at a concentration of 10,000 cells/cm^2^ (t = 0) and cultured up to 72 h. The cultures were imaged at 24, 48 and 72 h using a Nikon Eclipse-Ti inverted wide-field microscope (Nikon, Tokyo, Japan) equipped with a 20× air Nikon objective (NA 0.45, Plan-Fluor), an incubating chamber (Okolab, Naples, Italy) and a CCD ORCA R2 (Hamamatsu, Iwata City, Japan). In order to assess the proliferation rate on the chitosan membranes, the GFP-positive cells were quantified on different samples at 24 and 72 h using an ImageJ program (NIH, Bethesda, MD, USA), and their concentration was expressed as cells/mm^2^. At least 10 images/sample were analyzed. We performed *n* ≥ 3 independent experiments for each condition.

### 4.7. Immunostaining

RT4-SCs were grown for 3 days on FLAT, GR, ISO and SCA chitosan membranes, then fixed for 15 min in 4% paraformaldehyde in PBS at RT and processed as previously reported [31]. The cells were stained with primary antibodies in GDB buffer (0.2% BSA, 0.8 M NaCl, 0.5% Triton X-100, 30mM phosphate buffer, pH 7.4), containing phalloidin-Alexa647 (Invitrogen, Waltham, MA, USA, A22287; 1:40) to stain the actin fibers (F-actin), overnight at 4 °C. Primary antibodies: anti-N-Cadherin (N-Cad; BD Transduction Lab., Franklin Lakes, NJ, USA; 1:250, mouse). The samples were then washed and incubated with the appropriate AlexaFluor Plus555 conjugated secondary antibodies (Invitrogen; 1:150) in GDB for 1 h at RT. After the washing, the samples were mounted using Fluoroshield histology mounting medium with 4′,6-diamidino-2-phenylindole (DAPI) (Sigma-Aldrich, F6182).

### 4.8. Confocal Imaging and Morphological Analysis

Confocal images were acquired using a laser scanning confocal microscope TCS SP2 (Leica Microsystems, Wetzlar, Germany) with a 40× oil objective using 3 laser lines (488, 561 and 647 nm). Each reported confocal image was obtained from a z-series (the stack-depth was within 10 µm; steps = 1 µm). The resulting z-stack was processed using ImageJ software (Ver. 1.52t, NIH, Bethesda, MD, USA) into a single image using the “z-project” and “Max intensity” options. The confocal settings were kept the same for all of the scans when the fluorescence intensity was compared.

The confocal images of the F-actin were used to evaluate the single-cell and protrusion morphology by ImageJ (NIH). The cell contours were drawn by the “Polygon selection” tool and processed by the “Measurement” tool (with the options “Area”, “Fit ellipse” and “Feret’s diameter”). The orientation of the patterns was measured by the “Angle tool” of ImageJ; for the FLAT membranes, a random direction was chosen. The parameters measured in this analysis were: the cell area (μm^2^); the cell aspect ratio (the ratio between the length of the major axis and the minor axis for the best-fitted ellipse of the cell); and the cell alignment angle (the angles were calculated as the absolute value of the difference between the orientation angle of the grating and of the cell/nuclear major axis). At least 25 cells per sample were analysed.

The cell protrusions’ contours were also drawn using the “Polygon selection” tool and analysed using the “Measurement” tool (with the options “Area” and “Mean gray value”). With almost all of the cells being bipolarized on the micro-patterns, the parameters measured in this analysis were: the ratio of the protrusions’ area (the ratio between the area of the two cells’ protrusions, the bigger one over the minor one), as the parameter of the cell-end asymmetry; and the ratio of the F-actin intensity between the two cell protrusions (the ratio between the F-actin mean intensity of the bigger protrusion and the minor one, for each cell), as the parameter of the cell-end contractility asymmetry. Each protrusion’ area was drawn with a fixed length of 15 µm (i.e., along the cell and underlying the pattern’s main axis, in the soma direction), with the aim to analyze the same segment in all of the cells and to avoid soma compartment. At least 12 cells were analyzed per sample.

The SC cytoskeleton and the cell–cell junctions’ organization was quantified by analyzing the F-actin and N-Cad fluorescence signal, respectively, with the “Directionality” tool of the software FIJI (NIH, Bethesda, MD, USA, Ver. 1.52t, http://fiji.sc/Fiji), similarly to [31]. This plugin returned a directionality histogram by exploiting image fast Fourier transform (FFT) algorithms: isotropic images generate a flat histogram, whereas oriented images give a peaked histogram. These histograms were finally fitted by Gaussian curves that returned two parameters, dispersion and directionality (the standard deviation and the center of the Gaussian curve, respectively), the first representing the degree of orientation of the image, the second representing the direction in which it is oriented (here normalized to the underlying pattern orientation direction). We analyzed at least 5 fields/sample; the image dimensions were kept fixed to 188 × 188 µm^2^.

For the nucleus analysis, the nuclei perimeter was drawn with the “Wand Selection” tool (which draws an ellipse around the nuclei) and processed with the “Measurement” tool in ImageJ. The nuclear aspect ratio and the alignment angle (as above for the cells) were calculated in [45]. We analyzed at least 30 nuclei/sample. We performed *n* ≥ 3 independent experiments for each condition.

### 4.9. Single Cell Migration Experiments

RT4-SCs were seeded on FLAT, GR, ISO and SCA chitosan films at a concentration of 10,000 cells/cm^2^. After 24 h, living-cell imaging was performed using a 20× air Nikon objective (N.A. 0.45, PlanFluor) and an Eclipse-Ti inverted wide-field microscope (Nikon, Japan) equipped with a perfect focus system, an incubating chamber (Okolab, Italy) and a CCD ORCA R2 (Hamamatzu, Japan). Bright-field and fluorescent images were acquired for 24 h, sampling every 10 min. The tracks were traced on GFP-fluorescence images and analyzed with the ImageJ manual tracking plugin MTrack, as in [31]. The coordinates of single cells (at least 15 cells/sample), as a function of time, were extracted and analyzed using a custom-made Matlab script. The following parameters were measured: (1) The migration steps (dS, corresponding to the cell motion calculated in 15 min), and the step vectors were analyzed along two directions; dS was considered parallel (dS║) if the angle between the step and the grating was between 0° and 15°, while it was considered perpendicular (dS⊥) for angle between 75° and 90°. The amount of parallel or perpendicular dS was reported as a percentage over the dS total number. (2) The cell displacement (the distance, in µm, from the origin after 15 h). (3) The average cell speed (V, in µm/h): as previously for dS, the average speed was also quantified as parallel (V║) and perpendicular (V⊥). (4) The persistence (the percentage of parallel steps in the predominant direction of the migration of the cells), as an indicator of the probability of the cell to persist in one direction of migration instead of reverting, along the main axis of the pattern. At least 15 cells per condition were tracked for each substrate. We performed *n* = 3 independent experiments for each condition.

### 4.10. Wound Healing Experiments

The wound healing measurements were performed on monolayers of RT4-SCs, on FLAT, GR, ISO and SCA membranes. The cells were seeded on chitosan films in two monolayers separated by a gap (average size Wmean = 388 ± 76 µm, mean ± SD) perpendicular to the pattern (or along a random direction in the case of the FLAT membranes), thanks to PDMS culture-inserts with 2 wells (81176, IBIDI GMBH, Gräfelfing, Germany), at a concentration of 80,000 cells/cm^2^. The next day, the PDMS culture-inserts were removed (t = 0) and images were acquired using a Nikon-Ti wide field microscope (see above) using a 20× objective (Nikon). The wound healing time series were recorded by time-lapse microscopy, and the wound area was measured after 20 h. The percentage of the area closure was finally reported. At least *n* = 3 independent experiments were performed for each substrate type.

### 4.11. Statistical Analysis

All of the experiments were repeated at least three times independently for each condition. The data were reported as the average value ± the standard error of the mean (mean ± SEM), unless differently stated. The data were statistically analyzed using GraphPad Prism 5.00 commercial software (GraphPad Software, San Diego, CA, USA). The mean values obtained in each repeated experiment were assumed to be normally distributed about the true mean. A one-way ANOVA with Bonferroni’s multiple comparison test analysis was used, unless differently stated, to compare the substrates. Statistical significance refers to results where *p* < 0.05 was obtained.

## 5. Conclusions

We here demonstrated that it is possible to obtain chitosan films with a micro-structured pattern using a soft-lithography technique that does not impact on the biological and mechanical properties of the material. This production by the solvent casting of chitosan biocompatible substrates is simple (e.g., no clean room facilities are needed for the process itself, while any original mold can also be purchased easily on the market) and cost-effective (e.g., high mold reuse).

Three directional micro-patterns (GR, ISO, SCA) were created, with increasing levels of asymmetry, and the better performing pattern was the most asymmetrical one, SCA. Not only did SCA induce a further-oriented migration and inhibited direction reversal, it also improved the wound healing response and in vitro cell proliferation. The higher freedom and asymmetry in the cell shaping allowed by the SCA pattern increased the cell migration and gave a preferential migration direction to the cells, and these are important issues in nerve regenerative applications.

This knowledge represents a useful tool for tissue engineering applications. Schwann cell migration and alignment into the injury site is a fundamental step for the following nerve regrowth. With simple geometrical principles, it is possible to guide the Schwann cell migration into the wound, enhancing their migration speed in the direction that is helpful for tissue regrowth. These results provide important information on how specific topographical features can be exploited for tissue engineering applications, and for the fabrication of new scaffolds that have better performances in peripheral nerve regeneration.

## Supplementary Materials

The following are available online at https://www.mdpi.com/article/10.3390/ijms22157901/s1. Figure S1: Representative bright field and fluorescent images of RT4 Schwann cells cultured on ISO, SCA and FLAT chitosan membranes, at 72 h. Figure S2: Merged confocal images of RT4-Schwann cells cultured on GR, ISO, SCA and FLAT chitosan membranes: actin fibers (red), N-Cadherin (yellow), nuclei (blue); scale bar = 20 µm. Figure S3: Average cell speed (µm/h) in the perpendicular direction (±15°) with respect to the pattern’s main direction.
