# Supplementary material for: Chitosan Micro-Grooved Membranes with Increased Asymmetry for the Improvement of the Schwann Cell Response in Nerve Regeneration
Source: Int J Mol Sci. 2021 Jul 23;22(15):7901. doi: 10.3390/ijms22157901 (PMC8348329; doi:10.3390/ijms22157901)
Supplement: Supplementary file 1 [file ijms-22-07901-s001.zip › ijms-1275805-supplementary.pdf]

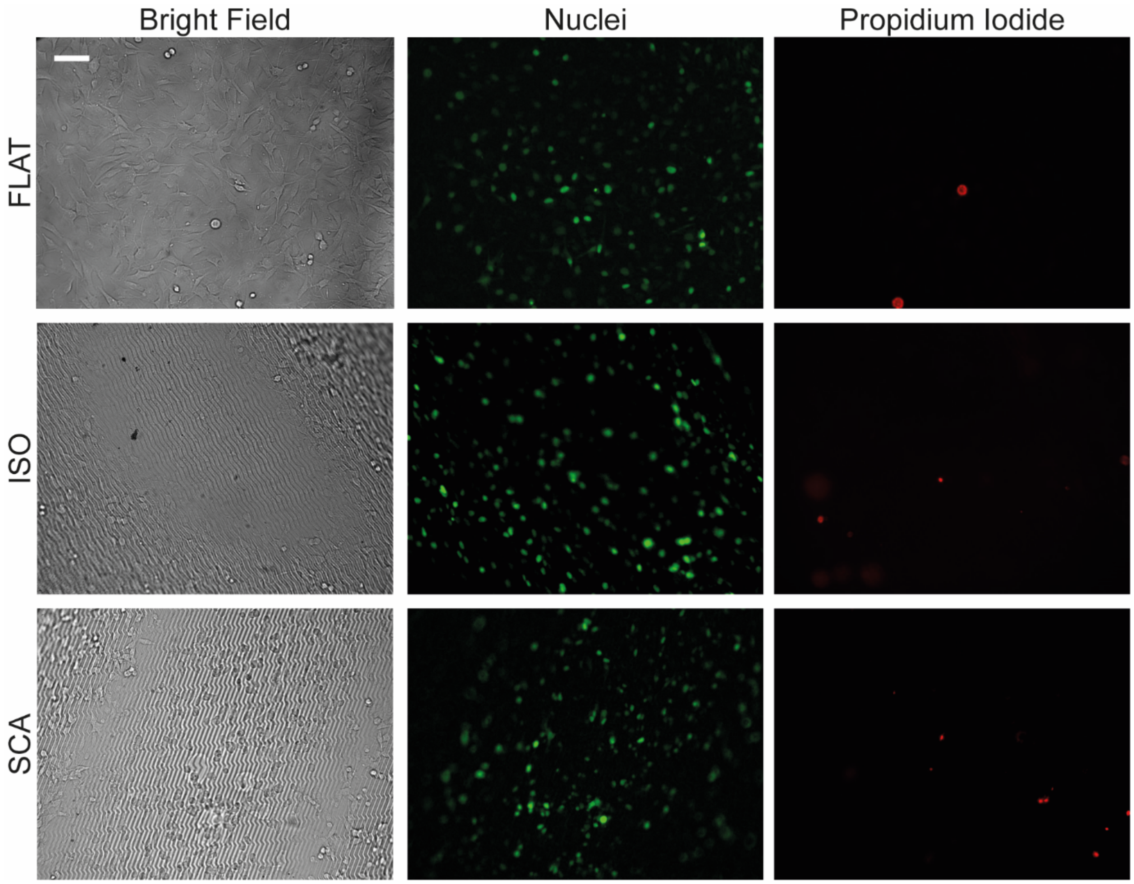

Figure S1. Representative bright field and fluorescent images of RT4 Schwann cells cultured on FLAT, ISO, and SCA chitosan membranes, at 72 hours. From left to right: bright field, GFP-positive cells (nuclei in green) and Propidium Iodide-positive dead cells (in red). Scale bar = 70µm (the scale bar is the same for all the images in this panel).

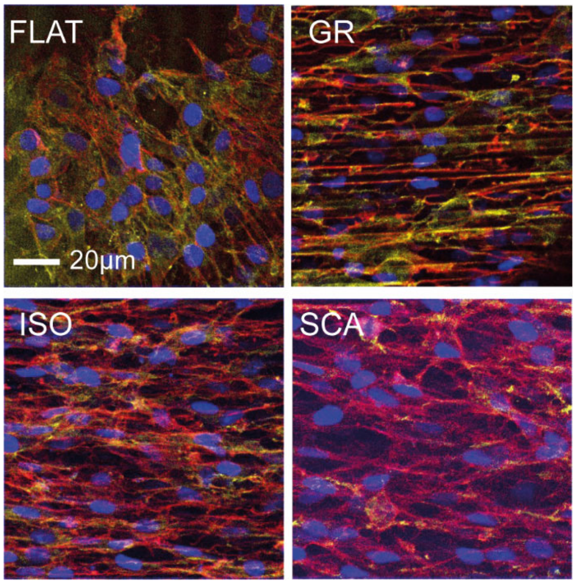

Figure S2. Merged channels of confocal images of RT4-Schwann cells cultured on GR, ISO, SCA and FLAT chitosan membranes: actin fibers (red), N-Cadherin (yellow), nuclei (blue); scale bar = 20µm.

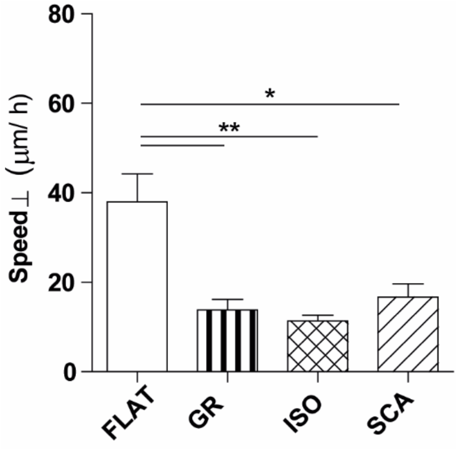

Figure S3. Average cell speed (µm/ h) in the perpendicular direction (± 75-90°) in respect to pattern main direction: * $P < 0.05$, ** $P < 0.01$, One-Way ANOVA, Bonferroni's test.
